# Supplementary figures and images for: Molecular dynamics of the matrisome across sea anemone life history
Source: eLife. 2025 Oct 23;14:RP105319. doi: 10.7554/eLife.105319 (PMC12549020; doi:10.7554/eLife.105319)

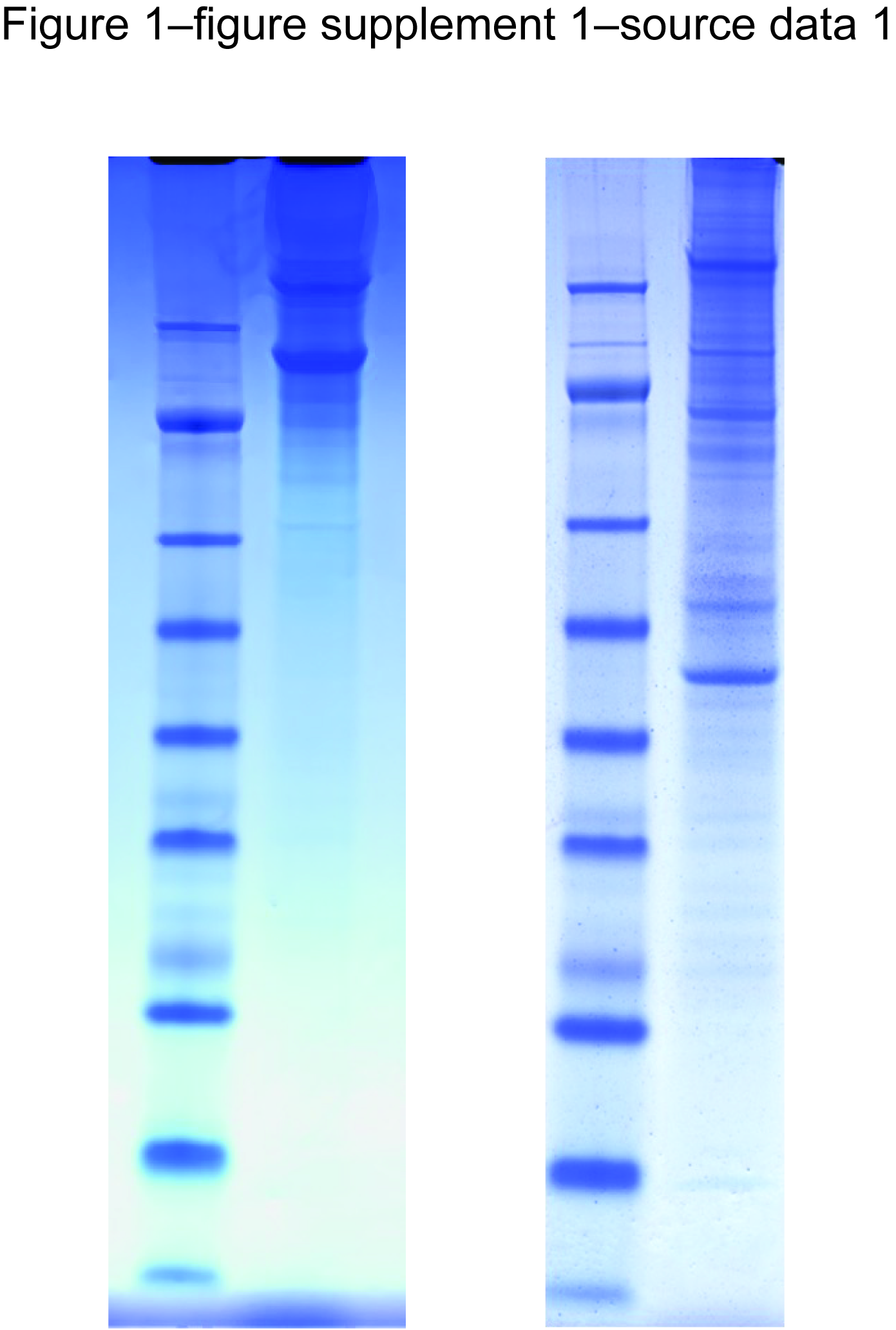

Supplement: Figure 1—figure supplement 1—source data 1. [file elife-105319-fig1-figsupp1-data1.zip › Fig. S1-Source data 1.tif]

Figure 1–figure supplement 1–source data 2

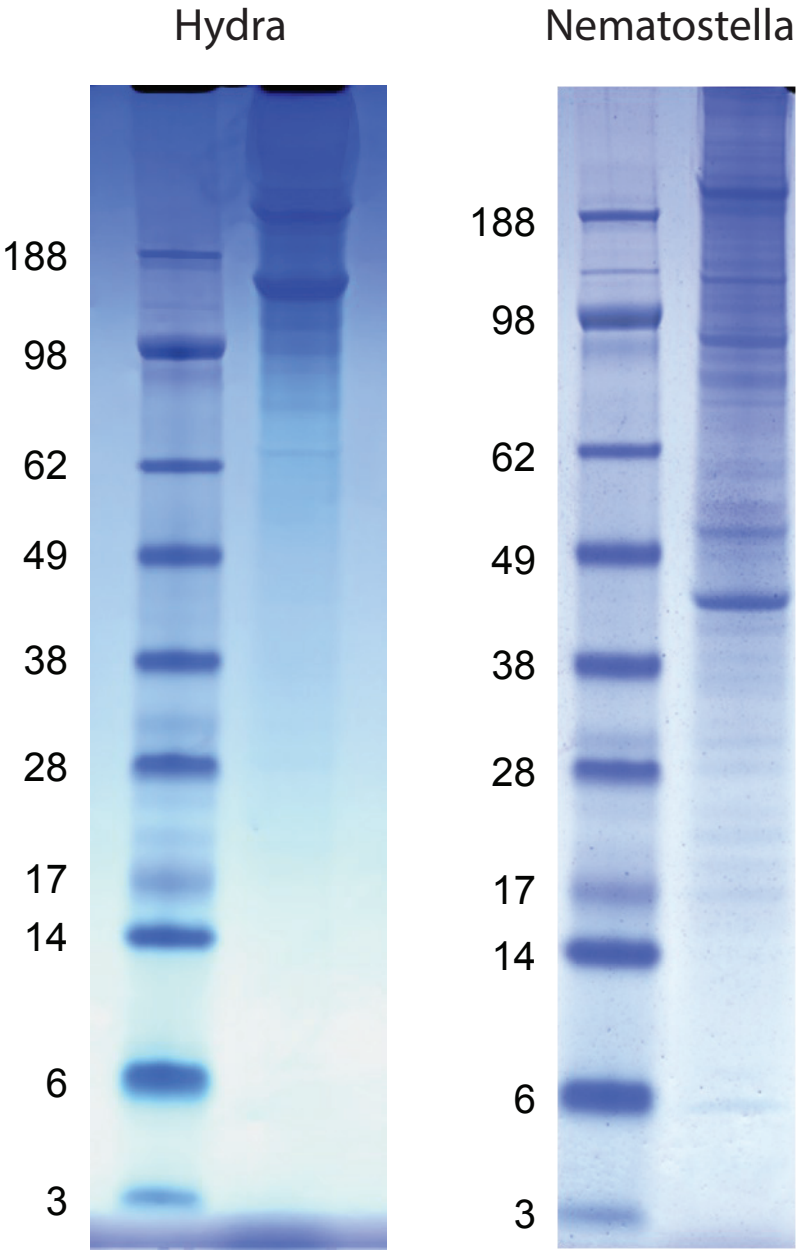

Supplement: Figure 1—figure supplement 1—source data 2. [file elife-105319-fig1-figsupp1-data2.pdf]
